# Supplementary material for: Application of Quantitative Computed Tomographic Perfusion in the Prognostic Assessment of Patients with Aneurysmal Subarachnoid Hemorrhage Coexistent Intracranial Atherosclerotic Stenosis
Source: Brain Sci. 2023 Apr 6;13(4):625. doi: 10.3390/brainsci13040625 (PMC10137244; doi:10.3390/brainsci13040625)
Supplement: Supplementary file 1 [file brainsci-13-00625-s001.zip › brainsci-2306759-SI.pdf]

Supplementary Table S1. Comparisons of baseline characteristics of included and excluded patients with aneurysmal subarachnoid hemorrhage and internal carotid artery stenosis.

Characteristics	Patients with CTP (Included)	Patients without CTP (Excluded)	P value
Sample size	192	41	
Age, yrs, mean±SD	59.34±10.77	59.73±11.45	0.836
Female sex	110 (57.3)	24 (58.5)	0.884
Prior medical history			
Smoking	29 (15.1)	17 (41.5)	<0.001
Drinking	15 (7.8)	8 (19.5)	0.023
Hypertension	113 (58.9)	30 (73.2)	0.087
Hyperlipidemia	10 (5.2)	4 (9.8)	0.266
Diabetes mellitus	16 (8.3)	5 (12.2)	0.443
Heart disease	23 (12.0)	6 (14.6)	0.640
Prior infarction	15 (7.8)	10 (24.4)	0.002
Aneurysm characteristics			
Posterior circulation	27 (14.1)	6 (14.6)	0.934
Early seizures			
Early loss of consciousness			
Acute hydrocephalus	83 (43.2)	13 (31.7)	0.174
Parent artery stenosis	88 (45.8)	22 (53.7)	0.362
Neurological score			
H-H grade 4-5	35(18.2)	6 (14.6)	0.583
WFNS grade 4-5	59 (30.7)	10 (24.4)	0.420
mFS grade 3-4	130 (67.7)	24 (58.5)	0.260
Graeb score 5-12	21 (10.9)	6 (14.6)	0.502
SEBES score 3-4	79 (41.1)	15 (36.6)	0.589
Treatment modality			0.466
Surgical clipping	101 (52.6)	19 (46.3)	
Endovascular treatment	91 (47.4)	22 (53.7)	
In-hospital complications			
Delayed cerebral ischemia	67 (34.9)	15 (36.6)	0.837
Hypoproteinemia	64 (33.3)	9 (22.0)	0.154
Stress ulcers bleeding	37 (19.3)	8 (19.5)	0.972
Anemia	78 (40.6)	13 (31.7)	0.288
Deep venous thrombosis	23 (12.0)	4 (9.8)	0.686
Pneumonia	91 (47.4)	18 (43.9)	0.684

Abbreviations: CTP, computed tomography perfusion; DCI, delayed cerebral ischemia; H-H, Hunt and Hess; mFS, modified Fisher Scale; SD, standard deviation; SEBES, Subarachnoid hemorrhage Early Brain Edema Score; WFNS, World Federation of Neurosurgical Societies.

Supplementary Table S2. The Sensitivity, Specificity, Accuracy, Positive Predictive Value (PPV), and Negative Predictive Value (NPV) of different models.

Model	Sensitivity	Specificity	Accuracy	PPV	NPV
SAHIT	0.913	0.381	0.797	0.840	0.5512
SAHIT+CTP	0.940	0.452	0.833	0.860	0.679
TAPS	0.907	0.429	0.802	0.850	0.563
TAPS+CTP	0.933	0.429	0.823	0.854	0.643

Abbreviations: CTP, Computed Tomography Perfusion; SAHIT, Subarachnoid Hemorrhage International Trialists; TAPS, Tiantan Aneurysmal Subarachnoid Hemorrhage Prognostic Scoring System.

Supplementary Table S3. The Performance of different models after the random under-sampling procedure.

Model	AUC (95%CI)	Sensitivity	Specificity	Accuracy	PPV	NPV
SAHIT	0.777 (0.702-0.852)	0.720	0.762	0.729	0.915	0.432
SAHIT+CTP	0.779 (0.699-0.860)	0.740	0.667	0.724	0.888	0.418
TAPS	0.700 (0.608-0.791)	0.747	0.643	0.724	0.882	0.415
TAPS+CTP	0.701 (0.604-0.798)	0.693	0.738	0.703	0.904	0.403

Abbreviations: AUC, area under the curve; CTP, computed tomography perfusion; NPV, negative predictive value; PPV, positive predictive value; SAHIT, Subarachnoid Hemorrhage International Trialists; TAPS, Tiantan Aneurysmal Subarachnoid Hemorrhage Prognostic Scoring System.
